# Case Report: Pregnancy-associated fulminant type 1 diabetes superimposed on insulin-treated gestational diabetes with intrauterine fetal demise

**DOI:** 10.3389/fendo.2026.1994868

**Published:** 2026-10-30

**Authors:** Dexia Li, Leweihua Lin, Zhenshu Gao, Danhong Lin, Jiahuan Zhang, Huibiao Quan

**Affiliations:** 1 Department of Endocrinology, Hainan General Hospital, Affiliated Hainan Hospital of Hainan Medical University, Haikou, China; 2 Department of Hematology, The Second Affiliated Hospital of Hainan Medical University, Haikou, China

**Keywords:** case report, diabetic ketoacidosis, fulminant type 1 diabetes, gestational diabetes mellitus, intrauterine fetal demise

## Abstract

Pregnancy-associated fulminant type 1 diabetes (FT1DM) is characterized by abrupt loss of endogenous insulin secretion and rapid progression to diabetic ketoacidosis (DKA). Recognition may be particularly challenging when FT1DM develops in a pregnancy already treated with insulin for gestational diabetes mellitus (GDM), because sudden glycemic deterioration may be attributed to increasing gestational insulin resistance, inadequate insulin dosing, or treatment interruption. We report a 24-year-old woman with insulin-treated GDM who developed dizziness, nausea, and vomiting at 33 weeks of gestation after one scheduled basal insulin dose was omitted. At a referring hospital, plasma glucose was 19.14 mmol/L (345 mg/dL) and urinary ketones were 3 +. Fetal cardiac activity was reportedly present initially but was absent the following day, when intrauterine fetal demise was confirmed. After transfer, plasma glucose was 20.77 mmol/L (374 mg/dL), beta-hydroxybutyrate 6.63 mmol/L (69.0 mg/dL), arterial pH was 7.235, and bicarbonate was 6.00 mmol/L (6.00 mEq/L), confirming persistent severe DKA; glycated hemoglobin was 6.1% (43 mmol/mol). Fasting and stimulated C-peptide concentrations remained profoundly reduced after metabolic stabilization, supporting the diagnosis of pregnancy-associated FT1DM. DKA improved with intravenous fluids, insulin, electrolyte replacement, and close biochemical monitoring, after which labor was induced and a stillborn female fetus was delivered. Repeated glucose and C-peptide measurements during long-term follow-up demonstrated persistent near-complete endogenous insulin deficiency. This case highlights the risk of diagnostic anchoring in insulin-treated GDM. Abrupt gastrointestinal symptoms or unexpected glycemic deterioration should prompt immediate assessment for ketosis and acidosis rather than being attributed solely to gestational insulin resistance or an isolated interruption of insulin treatment.

## 1 Introduction

Fulminant type 1 diabetes mellitus (FT1DM) is characterized by abrupt pancreatic beta-cell failure, rapid progression to ketosis or diabetic ketoacidosis (DKA), marked hyperglycemia with a relatively modest increase in glycated hemoglobin (HbA1c), and profoundly reduced endogenous insulin secretion (1, 2). When FT1DM develops during pregnancy or within 2 weeks after delivery, it is generally classified as pregnancy-associated FT1DM and is associated with a particularly high risk of adverse fetal outcomes (3).

FT1DM occurring in a pregnancy already complicated by gestational diabetes mellitus (GDM) presents an additional diagnostic challenge. Several cases of GDM-associated FT1DM have been reported, including pregnancies requiring insulin treatment (4–6). A 2024 case series and literature review summarized seven GDM-associated FT1DM cases, all with fetal survival (6), whereas fetal death has been described in pregnancy-associated FT1DM without preceding GDM (7). In this setting, abrupt worsening of glycemia may initially be attributed to increasing pregnancy-related insulin resistance, insufficient insulin dosing, or treatment interruption. Such diagnostic anchoring may delay recognition of rapidly developing severe endogenous insulin deficiency and DKA.

We report a woman with insulin-treated GDM who developed pregnancy-associated FT1DM with severe DKA and intrauterine fetal demise in the third trimester. Serial glucose and C-peptide measurements during the acute phase and long-term follow-up provided objective evidence of profound and persistent endogenous insulin deficiency. This case underscores the importance of reconsidering the diagnosis when the clinical course of established GDM changes abruptly.

## 2 Case description

A 24-year-old woman, gravida 2 para 1, with no significant medical history or family history of diabetes underwent a 75-g oral glucose tolerance test at 24 weeks and 5 days of gestation. Plasma glucose concentrations were 4.98 mmol/L (90 mg/dL) fasting, 9.12 mmol/L (164 mg/dL) at 1 hour, and 8.63 mmol/L (155 mg/dL) at 2 hours; glycated hemoglobin (HbA1c) was 5.5% (37 mmol/mol). The 2-hour glucose concentration met the diagnostic threshold for GDM. Dietary and exercise counseling was initially provided. Because fasting glucose remained 5.8–6.1 mmol/L (104–110 mg/dL), insulin detemir 14 units nightly was initiated at 28 weeks and 2 days. Thereafter, fasting glucose was approximately 5 mmol/L (90 mg/dL), and 2-hour postprandial glucose was approximately 6–7 mmol/L (108–126 mg/dL).

At 33 weeks of gestation, one scheduled basal insulin dose was not administered. The following day, the patient developed dizziness, nausea, and vomiting and presented to a local hospital. Random plasma glucose was 19.14 mmol/L (345 mg/dL), with urinary ketones 3+ and glucose 2 +. Intravenous fluids and continuous intravenous insulin were initiated for suspected DKA. Fetal cardiac activity was reportedly present at initial presentation. The following day, fetal heart sounds were absent, and ultrasonography confirmed intrauterine fetal demise. The patient was subsequently transferred to our hospital.

On arrival, her temperature was 36.5 °C, pulse was 94 beats/min, respiratory rate was 20 breaths/min, and blood pressure was 100/63 mmHg. She was alert but fatigued, and cardiopulmonary examination was unremarkable. Despite treatment initiated before transfer, plasma glucose was 20.77 mmol/L (374 mg/dL), beta-hydroxybutyrate was 6.63 mmol/L (69.0 mg/dL), arterial pH was 7.235, bicarbonate was 6.00 mmol/L (6.00 mEq/L), and base excess was −18.5 mmol/L (−18.5 mEq/L), confirming persistent severe DKA. HbA1c was 6.1% (43 mmol/mol). The clinical course is summarized in **Figure 1**, and key metabolic and beta-cell function findings are presented in **Table 1**.

**Figure 1:**
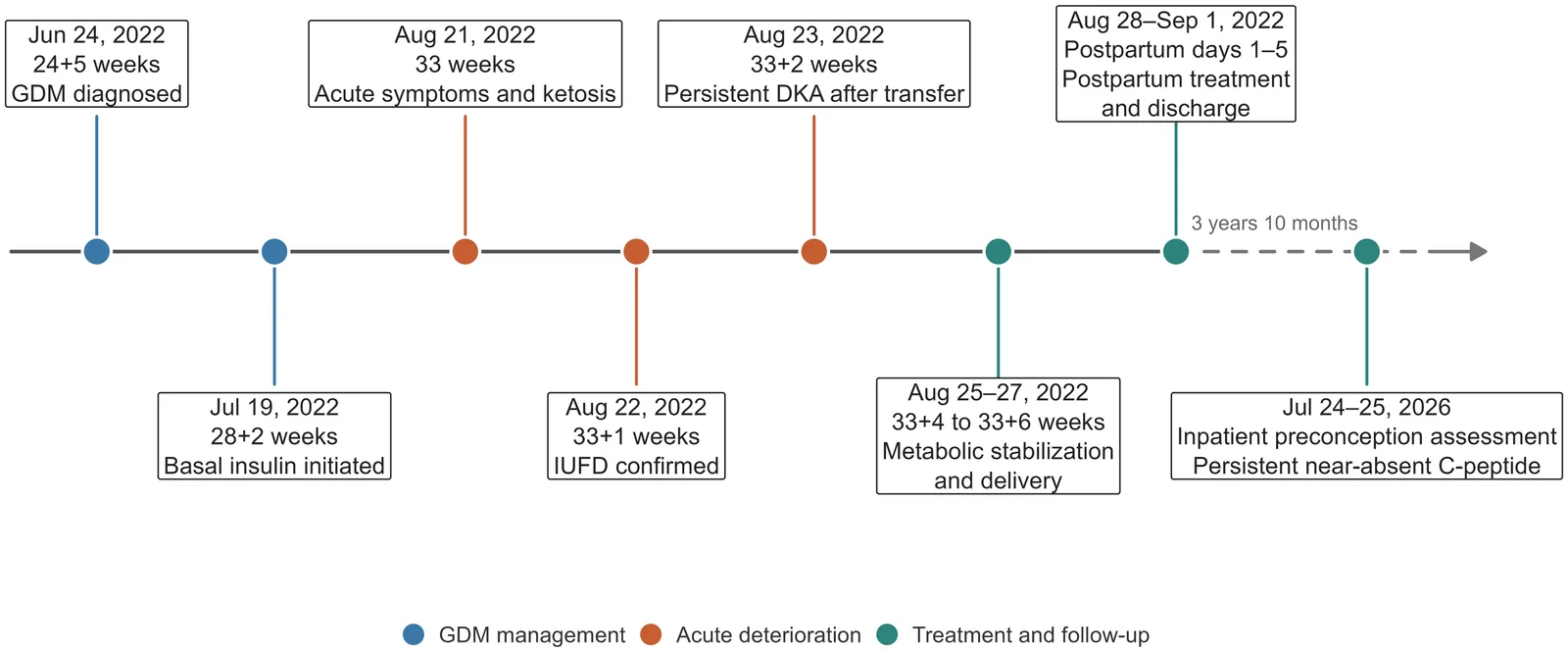
Clinical timeline from gestational diabetes diagnosis to pregnancy-associated fulminant type 1 diabetes, diabetic ketoacidosis, intrauterine fetal demise, and long-term follow-up. GDM was diagnosed at 24 weeks and 5 days, and basal insulin was initiated at 28 weeks and 2 days. Acute symptoms and ketosis developed at 33 weeks, followed by intrauterine fetal demise and persistent DKA. After metabolic stabilization, delivery, and postpartum treatment, long-term reassessment at 3 years and 10 months demonstrated persistent near-absent C-peptide. The timeline is schematic and not drawn to scale. DKA, diabetic ketoacidosis; FT1DM, fulminant type 1 diabetes mellitus; GDM, gestational diabetes mellitus; IUFD, intrauterine fetal demise.

**Table 1:** Key metabolic and beta-cell function findings across the clinical course.

Parameter	GDM diagnosis	Persistent DKA after transfer	Substantial DKA correction	Postpartum stabilization	Long-term follow-up 1	Long-term follow-up 2
**Timing**	24 + 5 weeks	33 + 2 weeks	Hospital day 3; 33 + 4 weeks	Postpartum day 3	3 years 5 months after discharge	3 years 10 months after discharge
**Date**	June 24, 2022	August 23, 2022	August 25, 2022	August 30, 2022	February 6, 2026	July 24–25, 2026
75-g OGTT glucose, 0/60/120 min, mmol/L (mg/dL)	4.98 (90)/9.12 (164)/ 8.63 (155)	—	—	—	—	—
HbA1c, % (mmol/mol)	5.5 (37)	6.1 (43)	—	—	9.7 (83)	8.9 (74)
Serum glucose, mmol/L (mg/dL)	—	20.77 (374)	12.38 (223)	—	—	7.10 (128)
Beta-hydroxybutyrate, mmol/L (mg/dL)	—	6.63 (69.0)	0.63 (6.6)	—	—	0.08 (0.8)
Arterial pH	—	7.235	7.378	—	—	—
Bicarbonate, mmol/L (mEq/L)	—	6.00 (6.00)	13.50 (13.50)	—	—	—
0-min glucose, mmol/L (mg/dL)	—	—	12.80 (230)	4.00 (72)	5.68 (102)	7.51 (135)^c^
0-min C-peptide, nmol/L (ng/mL)	—	0.073 (0.22)^a^	0.039 (0.12)	0.003 (0.009)	0.0033 (0.01)^b^	<0.003 (<0.009)^c^
120-min glucose, mmol/L (mg/dL)	—	—	11.40 (205)	7.80 (140)	17.56 (316)	4.55 (82)^c^
120-min C-peptide, nmol/L (ng/mL)	—	—	0.029 (0.09)	0.003 (0.009)	<0.0033 (<0.01)^b^	<0.003 (<0.009)^c^

Values are presented in SI units followed by conventional units in parentheses, except HbA1c, which is presented as percentage followed by mmol/mol. Measurements after transfer were obtained after intravenous fluids and insulin had been initiated at the referring hospital. On August 25, August 30, and February 6, the 0- and 120-min glucose and C-peptide values were paired; the routine serum glucose value of 12.38 mmol/L on August 25 was not paired with C-peptide. Em dashes indicate measurements not performed or not applicable.

^a^C-peptide was measured on August 24, 2022, during early DKA treatment without a simultaneous glucose measurement.

^b^C-peptide values originally reported as 0.01 and <0.01 ng/mL were converted to 0.0033 and <0.0033 nmol/L, respectively, using 1 ng/mL = 0.331 nmol/L.

^c^On July 24, 2026, fasting samples were obtained after an overnight fast; on July 25, 2026, 120-min samples were obtained 120 min after a routine breakfast during continuous subcutaneous insulin infusion. These measurements were therefore not obtained during the same meal test.

Diagnostic thresholds: GDM was diagnosed when one or more values on the 75-g oral glucose tolerance test met or exceeded 5.1, 10.0, and 8.5 mmol/L (92, 180, and 153 mg/dL) at 0, 60, and 120 min, respectively.

DKA, diabetic ketoacidosis; GDM, gestational diabetes mellitus; HbA1c, glycated hemoglobin; OGTT, oral glucose tolerance test; 0-min, fasting or baseline measurement; 120-min, 120 minutes after a meal.

## 3 Diagnostic assessment

Fasting C-peptide was 0.073 nmol/L (0.22 ng/mL) during early treatment of DKA. During subsequent paired testing, the 0-minute glucose concentration was 12.80 mmol/L (230 mg/dL) with a C-peptide concentration of 0.039 nmol/L (0.12 ng/mL), and the 120-minute glucose concentration was 11.40 mmol/L (205 mg/dL) with a C-peptide concentration of 0.029 nmol/L (0.09 ng/mL). After metabolic stabilization, fasting and 120-minute glucose concentrations were 4.00 and 7.80 mmol/L (72 and 140 mg/dL), respectively, while C-peptide was 0.003 nmol/L (0.009 ng/mL) at both time points. These findings demonstrated profound endogenous insulin deficiency after metabolic stabilization.

Glutamic acid decarboxylase and islet cell antibodies were within laboratory reference ranges. Insulin antibody concentrations were elevated after exposure to exogenous insulin and therefore could not be interpreted as evidence of spontaneous islet autoimmunity. Insulinoma-associated antigen-2 and zinc transporter 8 antibodies were not measured. Serum amylase was 96 U/L and lipase was 119.6 U/L; pancreatic ultrasonography showed no apparent abnormality.

According to the 2012 Japan Diabetes Society criteria, FT1DM is characterized by ketosis or ketoacidosis developing within approximately 7 days of symptom onset, plasma glucose ≥16.0 mmol/L (288 mg/dL), fasting C-peptide <0.10 nmol/L (0.30 ng/mL), and stimulated C-peptide <0.17 nmol/L (0.51 ng/mL) (2). The HbA1c criterion of <8.7% (72 mmol/mol) is not strictly applicable when glucose intolerance has already been diagnosed. The abrupt clinical course, severe DKA, and profound fasting and stimulated C-peptide depletion strongly supported pregnancy-associated FT1DM in this patient. Negative glutamic acid decarboxylase and islet cell antibodies were compatible with the fulminant phenotype but were not diagnostic. Because insulinoma-associated antigen-2 and zinc transporter 8 antibodies were unavailable, conventional autoimmune type 1 diabetes could not be completely excluded on immunologic grounds alone.

## 4 Therapeutic intervention

The patient received intravenous isotonic fluids, continuous intravenous insulin, potassium and other electrolyte replacement, cardiac monitoring, and frequent biochemical assessment. With treatment, beta-hydroxybutyrate decreased to 0.63 mmol/L (6.6 mg/dL), arterial pH increased to 7.378, and the metabolic disturbance substantially improved. After substantial correction of DKA, she was transferred to obstetric care. Glycemia was managed with continuous subcutaneous insulin infusion and frequent biochemical monitoring during induction of labor.

## 5 Follow-up and outcomes

After substantial correction of DKA, labor was induced, and a stillborn female fetus was delivered vaginally on August 27, 2022. At the family’s request, fetal autopsy and placental histopathological examination were not performed.

On postpartum day 1, plasma glucose increased to 17.4 mmol/L (313 mg/dL) and beta-hydroxybutyrate to 2.00 mmol/L (20.8 mg/dL). Intravenous insulin was restarted for recurrent ketonemia. Beta-hydroxybutyrate subsequently decreased to 0.06 mmol/L (0.6 mg/dL), without documented recurrent metabolic acidosis. After stabilization, continuous subcutaneous insulin infusion was discontinued, and the patient was transitioned to basal-bolus insulin therapy. She was discharged on insulin detemir 16 units once daily and insulin aspart 7, 7, and 8 units before breakfast, lunch, and dinner, respectively.

At follow-up 3 years and 5 months after discharge, the patient reported uninterrupted insulin treatment and no recurrent DKA requiring emergency care or hospitalization. HbA1c was 9.7% (83 mmol/mol). Fasting and 2-hour postprandial glucose concentrations were 5.68 and 17.56 mmol/L (102 and 316 mg/dL), respectively, with corresponding C-peptide concentrations of approximately 0.0033 and <0.0033 nmol/L (0.01 and <0.01 ng/mL). She reported plans for another pregnancy, and intensive glycemic optimization with multidisciplinary preconception assessment was recommended.

At follow-up 3 years and 10 months after discharge, she underwent inpatient preconception glycemic optimization with continuous subcutaneous insulin infusion. HbA1c was 8.9% (74 mmol/mol). Fasting and 120-minute glucose concentrations obtained on consecutive days were 7.51 and 4.55 mmol/L (135 and 82 mg/dL), respectively, while the corresponding C-peptide concentrations remained <0.003 nmol/L (<0.009 ng/mL) at both time points. These repeated long-term assessments demonstrated persistent near-complete endogenous insulin deficiency.

## 6 Discussion

This case illustrates an important diagnostic challenge when pregnancy-associated FT1DM develops in a woman already receiving insulin for GDM. Once GDM has been established, abrupt deterioration in glycemic control may be attributed to increasing gestational insulin resistance, inadequate insulin dosing, or treatment interruption. In the present patient, the omission of one scheduled basal insulin dose provided an especially plausible competing explanation. However, the rapid progression to severe DKA together with profound fasting and stimulated C-peptide depletion indicated a new state of near-complete endogenous insulin deficiency rather than uncomplicated worsening of GDM.

Previous reports confirm that FT1DM can occur in pregnancies complicated by GDM. Choy et al. described FT1DM in a woman with insulin-requiring GDM who ultimately delivered a healthy infant (4). A different patient from our institution was previously reported with pregnancy-associated FT1DM in the setting of GDM and also had a live birth (5). In addition, a four-case series described GDM-associated FT1DM with fetal survival in all reported pregnancies (6). By contrast, fetal death has been reported in pregnancy-associated FT1DM without preceding GDM (7). These reports indicate that the coexistence of GDM and FT1DM is not unique and that fetal outcomes are heterogeneous. The present case therefore broadens the documented clinical spectrum of GDM-associated FT1DM by describing severe DKA accompanied by intrauterine fetal demise in a patient already receiving insulin for GDM.

The diagnosis of FT1DM in this patient was supported primarily by the abrupt 2-day symptomatic course, severe DKA, and fasting and stimulated C-peptide concentrations far below the Japan Diabetes Society diagnostic thresholds (2). The HbA1c criterion also requires pregnancy-specific interpretation because increased red blood cell turnover may lower HbA1c during pregnancy, and HbA1c reflects integrated rather than acute glycemia (8). More generally, recent studies have emphasized that HbA1c may provide clinically relevant information beyond its use as a fixed diagnostic threshold (9, 10). Negative glutamic acid decarboxylase and islet cell antibodies were compatible with the fulminant phenotype but were not diagnostic. In addition, insulinoma-associated antigen-2 and zinc transporter 8 antibodies were not measured, and conventional autoimmune type 1 diabetes therefore could not be completely excluded on immunologic grounds alone. Nevertheless, the rapid clinical course and profound loss of endogenous insulin secretion strongly supported FT1DM.

Long-term follow-up was not required to establish the initial diagnosis. However, repeated glucose and C-peptide measurements more than 3 years after the acute event provided additional evidence that the severe insulin deficiency was persistent rather than the result of transient beta-cell suppression during DKA, pregnancy, or acute metabolic stress. The continued near-absence of fasting and stimulated C-peptide despite measurable glucose concentrations also argues against progressive GDM alone as an explanation for the profound beta-cell failure.

The omitted basal insulin dose warrants particular consideration. Interruption of basal insulin can accelerate ketogenesis in an insulin-deficient state and may therefore have contributed to the timing or severity of metabolic decompensation. However, a single missed dose cannot explain the profound fasting and stimulated C-peptide depletion documented after metabolic stabilization or the persistence of near-complete endogenous insulin deficiency during long-term follow-up. It is therefore more appropriately regarded as a possible precipitating factor for DKA rather than the cause of the underlying beta-cell failure.

Pregnancy itself predisposes to accelerated ketogenesis through increasing insulin resistance, enhanced lipolysis, continued fetoplacental glucose utilization, and reduced buffering capacity. Consequently, DKA may develop rapidly and sometimes at lower glucose concentrations than in nonpregnant patients (11). New nausea, vomiting, fatigue, or an abrupt worsening of glycemia during pregnancy should therefore prompt measurement of blood beta-hydroxybutyrate and assessment of acid-base status rather than being attributed automatically to pregnancy-related symptoms or worsening gestational insulin resistance. Immediate metabolic treatment and concurrent obstetric assessment are particularly important because maternal and fetal deterioration can occur within a short interval.

The intrauterine fetal demise in this patient occurred in close temporal association with severe maternal metabolic deterioration. Pregnancy-associated FT1DM has been associated with poor fetal outcomes (3), and DKA during pregnancy carries a substantial risk of fetal loss (12). Nevertheless, fetal autopsy and placental histopathological examination were unavailable in the present case. A direct causal relationship between maternal DKA and fetal death therefore cannot be established. Maternal acidemia, dehydration, electrolyte abnormalities, impaired uteroplacental perfusion, or another unrecognized fetal or obstetric factor may have contributed. Accordingly, the fetal demise should be interpreted as occurring in the setting of severe maternal DKA rather than as a definitively proven consequence of FT1DM.

The strengths of this report include documentation of the preceding GDM course and insulin treatment, the short interval between acute maternal symptoms and fetal deterioration, paired glucose and C-peptide measurements after metabolic stabilization, and repeated objective assessment of endogenous insulin secretion during long-term follow-up. Several limitations should also be acknowledged. C-peptide and islet autoantibodies were not measured before the acute event; insulinoma-associated antigen-2 and zinc transporter 8 antibodies were unavailable; insulin antibody measurements were obtained after exposure to exogenous insulin and therefore could not establish spontaneous islet autoimmunity; and fetal autopsy and placental histopathology were not performed. These limitations restrict complete characterization of the diabetes phenotype and prevent definitive determination of the cause of fetal death.

At long-term follow-up, glycemic control remained suboptimal, and the patient was planning another pregnancy. Preconception care should therefore include intensive glycemic optimization, assessment for diabetes-related complications, consideration of continuous glucose monitoring and appropriate insulin-delivery technology, and multidisciplinary management involving endocrinology and maternal-fetal medicine (8). Education should also emphasize uninterrupted basal insulin administration, sick-day management, home ketone testing when clinically indicated, and prompt medical assessment when gastrointestinal symptoms or unexpected hyperglycemia occur.

In conclusion, established insulin-treated GDM should not preclude reconsideration of the diagnosis when the clinical course changes abruptly. New gastrointestinal symptoms or unexpected glycemic deterioration should prompt immediate evaluation for ketosis and acidosis, particularly when the severity of metabolic deterioration is disproportionate to the preceding GDM course. Early recognition of pregnancy-associated FT1DM and simultaneous maternal metabolic and obstetric assessment are essential because severe maternal and fetal deterioration may develop rapidly.

## 7 Patient perspective

After this experience, I realized that I should not miss insulin injections and should seek medical attention promptly if I feel unwell. Daily insulin injections are inconvenient, and I need to pay more attention to my diet and blood glucose control. I now use insulin regularly, monitor my glucose, and adjust the dose according to the readings. I still hope to become pregnant again. I was admitted for glycemic optimization and have continued regular glucose monitoring after discharge. I would like to know what further preparations are needed before another pregnancy. My advice to other pregnant women with gestational diabetes using insulin is to seek medical attention promptly if unusual symptoms occur.

## Data Availability

The original contributions presented in this article are included in the article. Further inquiries may be directed to the corresponding author, subject to patient confidentiality and institutional requirements.
